# Multi-Objective Drug Molecule Optimization Based on Tanimoto Crowding Distance and Acceptance Probability

**DOI:** 10.3390/ph18081227

**Published:** 2025-08-20

**Authors:** Yuxin Wang, Cai Dai, Xiujuan Lei

**Affiliations:** School of Computer Science, Shaanxi Normal University, Xi’an 710119, China; wangyuxin@snnu.edu.cn (Y.W.); xjlei@snnu.edu.cn (X.L.)

**Keywords:** molecular optimization, evolutionary algorithm, drug discovery, Tanimoto distance

## Abstract

**Background**: Traditional molecular optimization methods struggle with high data dependency and significant computational demands. Additionally, conventional genetic algorithms often produce solutions with high similarity, leading to potential local optima and reduced molecular diversity, thereby limiting the exploration of chemical space. **Methods**: In order to address the above issues, this paper proposes an improved genetic algorithm for multi-objective drug molecular optimization (MoGA-TA). It uses the Tanimoto similarity-based crowding distance calculation and a dynamic acceptance probability population update strategy. The study employs a decoupled crossover and mutation strategy within chemical space for molecular optimization. The proposed crowding distance calculation method better captures molecular structural differences, enhancing search space exploration, maintaining population diversity, and preventing premature convergence. The dynamic acceptance probability strategy balances exploration and exploitation during evolution. Optimization continues until a predefined stopping condition is met. To assess MoGA-TA’s effectiveness, the algorithm is evaluated using metrics like success rate, dominating hypervolume, geometric mean, and internal similarity. **Results**: Experimental results show that compared to the comparative method, MoGA-TA performs better in drug molecule optimization and significantly improves the efficiency and success rate. **Conclusions**: The method described in this paper has been proven to be an effective and reliable method for multi-objective molecular optimization tasks.

## 1. Introduction

Optimizing drug molecules is a complex and crucial process focused on enhancing their physicochemical properties, biological activity, and selectivity [1,2]. This process constitutes a crucial element in drug discovery and necessitates ongoing optimization via the Design–Synthesis–Test–Analysis (DSTA) cycle. The DSTA cycle is an iterative strategy commonly used in drug optimization, which refers to the continuous improvement of the structure and properties of a candidate molecule through four steps: molecular design, synthesis, experimental testing, and analysis of results. The traditional drug discovery process is faced with long lead times, high costs, and high risks [3], especially in the huge chemical space—estimated to be about $10^{60}$ molecules—which makes finding molecules with desirable properties in such a large search space an extremely challenging task [4]. It is crucial to develop efficient lead compound optimization strategies to expedite the identification and development of novel drug candidates.

Computer-aided drug design (CADD) has significantly advanced molecular optimization methodologies, encompassing both generative model-based and evolutionary algorithm-based strategies. Nonetheless, generative model-based methods continue to encounter optimization efficiency issues owing to the absence of prior knowledge in drug molecular design and the constraints imposed by limited training datasets. Specifically, as deep generative models (DGMs) have flourished in the field of molecular design [5,6], models such as variational auto-encoders (VAEs) [7,8], recurrent neural networks (RNNs) [9], and generative adversarial networks (GANs) [10], with different molecular representations (e.g., SMILES strings or molecular graphs), have been widely used in molecular generation, attribute prediction, and optimization tasks [11,12]. These models help medicinal chemists efficiently design molecules and generate candidate molecules that match known good molecular properties by mapping discrete molecular representations (e.g., SMILES, molecular graphs) in chemical space to the continuous latent space and combining them with optimization models [13,14,15]. Despite the significant progress made by DGMs in the field of drug molecule optimization, the limitations of the available training data, the insufficient global search capability, and the lack of molecular data with multiple desirable attributes at the same time remain the major bottlenecks limiting the further development of these models [16,17,18]. Evolutionary algorithm-based methods typically require balancing multiple optimization objectives to achieve an optimal solution. Current approaches often employ an aggregation strategy to merge multiple objectives into a single objective, complicating the effective weighting of these objectives. Furthermore, most existing multi-objective optimization techniques are limited to optimizing two or three objectives, underscoring the importance of developing methods suitable for a greater number of objectives. Despite significant advancements in drug molecule optimization, addressing the scarcity of high-quality data and improving the balance of objectives in multi-objective optimization remain critical challenges in this domain.

Evolutionary algorithms (EAs) play a crucial role in drug molecule optimization, especially in multi-objective drug molecule design, where they show excellent performance [19,20]. These algorithms are adept at managing multiple optimization objectives concurrently, utilizing evaluation techniques such as non-dominated sorting and crowding distance for molecule selection, thereby efficiently identifying the optimal solution set along the Pareto frontier. The primary benefits of EAs include their robust global search capabilities and thorough exploration of intricate chemical landscapes, which facilitate exceptional performance in addressing molecular design challenges. EAs are recognized for their minimal reliance on extensive prior knowledge or large-scale training datasets, demonstrating significant flexibility in drug molecular design. Recent research, exemplified by studies like MolFinder and EvoMol [21,22], indicates that in certain scenarios, the efficacy of EAs not only matches but sometimes surpasses that of Deep Generative Models (DGMs) in specific tasks [23,24]. These studies emphasize the great potential of EAs in the field of drug molecule optimization, and in particular, they exhibit significant advantages in dealing with multi-objective optimization problems. Although EAs have been widely used in the development of ab initio drug discovery models, these models mainly focus on designing new molecules from scratch and are more focused on 2–3 objectives [25]. Therefore, there is still room for improving the optimization efficiency in generating structurally similar molecules with target properties based on existing lead compounds.

In the field of multi-objective evolutionary algorithms (MOEA), the non-dominated sorting genetic algorithm II (NSGA-II) has attracted much attention due to its high efficiency and excellent ability to maintain population diversity [26,27]. The algorithm selects individuals by non-dominated sorting and crowding calculations, thus maintaining population diversity and guiding the evolution of the population towards the Pareto front. In addition, the Tanimoto coefficient has an important role in the optimization process of drug molecules. Based on the principle of set theory, this coefficient measures the similarity of two sets by quantifying the ratio of their intersection to their concatenation [28]. Tanimoto coefficients are widely used in molecular similarity metrics and provide a powerful tool for tasks such as molecular clustering, classification, and retrieval [29]. Through these approaches, EAs are not only able to identify new molecules with desirable properties but also significantly enhance the efficiency and innovativeness of the drug discovery process, extend the exploration of chemical space, and enable fast and efficient searches.

We present an algorithm named MoGA-TA, which calculates Tanimoto similarity-based congestion distance and incorporates a dynamic population updating strategy to adjust the acceptance probability for molecular optimization. This algorithm integrates the multi-objective optimization capabilities of NSGA-II with the advantages of Tanimoto coefficient similarity measures. It is designed to optimize multiple objectives concurrently, including enhancing efficacy, reducing toxicity, increasing solubility, and improving other performance metrics. Through an iterative search process, it generates a set of non-dominated solutions that balance these objectives [30]. The Tanimoto crowding-based mechanism accurately captures structural differences between molecules, preserving diverse structures and guiding population evolution. The acceptance probability-based population update strategy enables broader exploration of chemical space during early evolution, balancing exploration and exploitation while preventing premature convergence to local optima. In later stages, this strategy effectively retains superior individuals, allowing the population to gradually converge towards the global optimum.

## 2. Results and Discussion

### 2.1. Benchmark Evaluation of MOGA-TA

To evaluate the performance of MoGA-TA, we compared MoGA-TA with NSGA-II and GB-EPI on six multi-objective molecular optimization tasks, of which the first five were from the GuacaMol benchmarking platform, and the sixth was aimed at optimizing the biological activity and drug-like properties [31,32,33].

#### 2.1.1. Test Tasks for Molecular Optimization

In this study, we use datasets from the ChEMBL database. The six benchmark tasks cover different molecular properties and optimization objectives. The six tasks are specified as follows:

- Task 1 (Fexofenadine): Tanimoto similarity (AP), TPSA, logP.
- Task 2 (Pioglitazone): Tanimoto similarity (ECFP4), molecular weight, number of rotatable bonds.
- Task 3 (Osimertinib): Tanimoto similarity (FCFP4), Tanimoto similarity (FCFP6), polar surface area (TPSA), logP.
- Task 4 (Ranolazine): Tanimoto similarity (AP), polar surface area (TPSA), logP, number of fluorine atoms.
- Task 5 (Cobimetinib): Tanimoto similarity (FCFP4), Tanimoto similarity (ECFP6), number of rotatable bonds, number of aromatic rings, CNS [34].
- Task 6 (DAP kinases): DAPk1, DRP1, ZIPk, QED, logP.

The objectives in the six optimization tasks include similarity between the molecule and the target drug, specific properties of the molecule, and physicochemical properties. As shown in Table 1, among the objectives of these benchmarking tasks, the similarity score is achieved by calculating the Tanimoto similarity of the fingerprint between the target molecule and the generated candidate molecule. This computational process was accomplished using models embedded in the RDKit software package (version 2022.09). The molecular fingerprints used therein include ECFP, FCFP, and atom pair fingerprint AP. For the obtained similarity scores and target scores, such as attributes, they are mapped to the [0, 1] interval after modification by the corresponding modifier functions in Table 1. Among them, the scoring functions can be calculated based on the SMILES string of the molecule or the molecule map, and the scores of TPSA and logP are calculated by the RDKit software package [35].

#### 2.1.2. Evaluation Metrics

In order to comprehensively evaluate each optimization task, we used four evaluation metrics: success rate, super volume, geometric mean, and internal similarity. Specifically, the success rate (SR) is the percentage of generated molecules that satisfy all the target conditions. The geometric mean measures the comprehensive performance of the generated molecules on multiple target attributes. We use an extended similarity index to calculate and track the internal similarity of evolving populations. In the optimization task, the conditions to be satisfied by the molecules are those that meet the thresholds given by the modification function. For example, for the optimization task Fexofenadine, molecules that satisfy the Tanimoto (AP) condition are those with similarity less than 0.8; molecules that satisfy the condition for the target TPSA are those with TPSA scores in the range of [80, 100]; and molecules that satisfy the condition for the target logP are those with logP values between [2, 6]. The hypervolume metric is a common measure of convergence and diversity of algorithms in multi-objective optimization, which calculates the hypervolume of the objective space dominated by the Pareto solution, and is mathematically defined as shown in Equation (1):

(1) $$HV=\delta \left(\overset{\left|S\right|}{\underset{i=1}{⋃}}v_{i}\right)$$

where HV represents the hypervolume [36]. δ is the Lebesgue measure used to evaluate the hypervolume. |S| represents the number of solutions in the non-dominated solution set (Pareto solutions). $v_{i}$ represents the hypervolume between the reference point and the *i*-th solution in the solution set, which is a hyperrectangle formed by the reference point (origin) and the *i*-th solution in the non-dominated solution set.

### 2.2. Comparisons on Test Tasks

In our experiments, we compared MoGA-TA with NSGA-II and GB-EPI [32,37]. To enhance the precision and impartiality of the experimental outcomes, identical benchmarking and experimental datasets were utilized for MoGA-TA, NSGA-II, and GB-EPI. Furthermore, the experimental configuration of the NSGA-II methodology was adopted, which entailed conducting 20 trials per optimization task, with a population size of 100 and 150 iterations. All experiments were conducted on a computing platform equipped with an RTX 3090 graphics card and an Intel(R) Xeon(R) Gold 6226R processor. The Python version used was 3.7.

**Figure 1:**
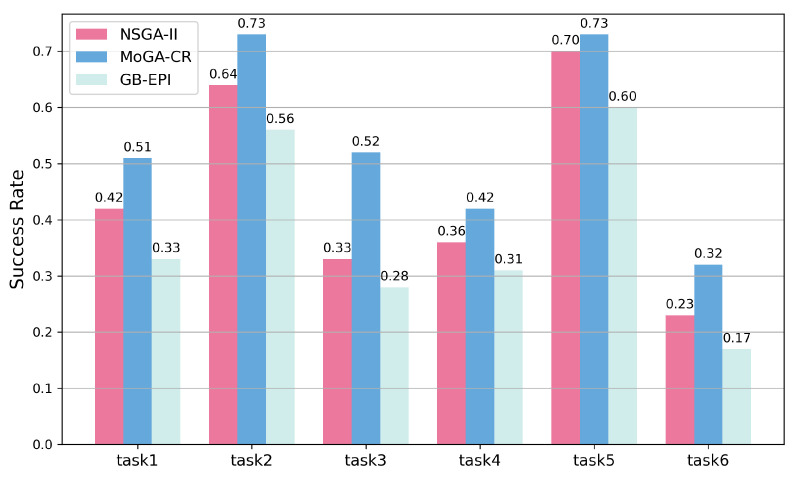
SR values for all comparison models across the six tasks.

First, Table 2 demonstrates the average values of the three methods on each evaluation metric [32]. In the six test tasks, the proposed MoGA-TA method significantly outperforms GB-EPI across all evaluation metrics and demonstrates superior overall performance compared to NSGA-II. Figure 1 illustrates the success rates of the three methods in the six optimization tasks. It shows that in tasks involving three optimization objectives, MoGA-TA achieves a success rate of 73%, which is 17% higher than GB-EPI and approximately 9% higher than NSGA-II. For tasks with four optimization objectives, such as Osimertinib optimization, MoGA-TA’s success rate exceeds NSGA-II by 19%. In the tasks involving five optimization objectives, the success rate of MoGA-TA for the optimization of multi-kinase inhibitors is still higher than the baseline method and 9% higher than NSGA-II. In addition, MoGA-TA has also achieved certain improvements in the hypervolume index, reflecting the improvement in the diversity, superiority, convergence, and algorithm performance of the generated molecules. In short, MoGA-TA has achieved better performance in all average values and has achieved significant improvements in the hypervolume and success rate indicators. It proves the effectiveness and feasibility of MoGA-TA in molecular optimization.

**Figure 2:**
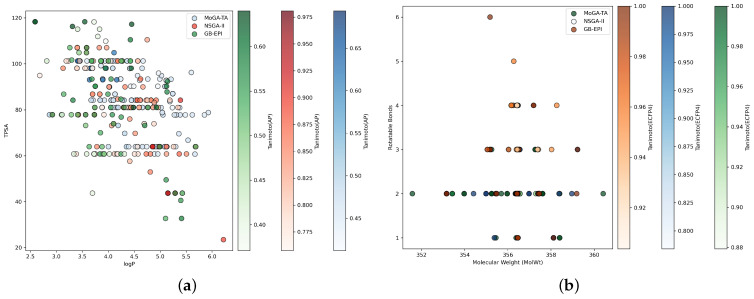
The distribution of molecules generated by MoGA-TA, GB-EPI, and NSGA-II in the chemical space when optimizing task 1 and task 2. The coordinate axes represent the corresponding attribute values. ((**a**) Task 1; (**b**) Task 2).

Second, in order to visualize the distribution of molecules generated by the two methods in the chemical space, Figure 2 shows the molecular spatial distribution of two representative benchmarks: Fexofenadine and Pioglitazone. As can be seen from Figure 2a, in the Fexofenadine optimization task, the molecules generated by the MoGA-TA method are more uniformly distributed and cover a wider range of the chemical space, suggesting that the MoGA-TA method has a better versatility in exploring the chemical space. In contrast, the molecular distribution of NSGA-II is more concentrated, and the number of molecules is dense in some areas, while the molecular distribution is sparse or very small in some areas. This suggests that this chemical region was not explored effectively or was insufficiently explored by NSGA-II. GB-EPI generated molecules with higher TPSA scores with a higher percentage of molecules, but fewer molecules within the TPSA score interval [80, 100], in addition to some molecules with lower TPSA scores. From the figure, it can be seen that the overall similarity of molecules generated by NSGA-II and GB-EPI is higher than that of molecules generated by MoGA-TA, whereas the similarity distribution of molecules generated by the MoGA-TA method is more uniform, which is more suitable for the generation of molecules with higher diversity and different similarity levels. Figure 2b shows that in optimizing the task Pioglitazone, the distribution of molecules generated by the NSGA-II method is more concentrated and fails to explore the chemical space effectively, while the GB-EPI method generates molecules with a relatively high proportion of molecules with a high number of rotatable bonds. However, too many rotatable bonds may lead to the instability of the molecular structure. Comparatively, most of the molecules generated by the MoGA-TA method have 1 or 2 rotatable bonds, and the generated molecules have a wider coverage and more dispersed distribution in the chemical space, which enables the exploration of more effective molecules. This suggests that the MoGA-TA method is able to explore different chemical spaces while maintaining the stability of the molecular structure. In addition, the molecules generated by the MoGA-TA method are more homogeneous in similarity distribution compared to GB-EPI, which helps to maintain the diversity of the generated molecules. In summary, the MoGA-TA method is able to discover molecules with large structural differences and exhibits a more uniform distribution in similarity scores, a property that is particularly prominent in multi-objective optimization tasks.

The molecular optimization method MoGA-TA, as presented in this paper, demonstrates superior performance as an optimization model. It excels particularly in tasks related to molecular structure optimization and multi-objective molecular property optimization. The exceptional performance of MoGA-TA can be attributed to its congested distance calculation method and dynamic acceptance probability strategy. These strategies enhance the ability to discern structural differences between molecules and effectively manage the balance between exploration and exploitation. Through dynamic adjustment of the acceptance probability, the algorithm introduces necessary randomness, preventing premature convergence to local optima and fostering the generation of diverse molecular structures.

Finally, this paper uses indicators such as success rate and maximum mean to show that the method can effectively improve the efficiency of generating molecules while meeting the optimization requirements when performing multi-objective optimization tasks. By visualizing the distribution of generated molecules in chemical space, it is verified that this method can explore a wider range of chemical space during the molecular optimization process. The experimental results show that in the optimization task of this paper, MoGA-TA can achieve relatively good results in the optimization of a given task.

### 2.3. Ablation Experiment

The ablation study focuses on evaluating two key aspects of population updating strategies: dynamically adjusted acceptance probabilities based on the number of iterations and congestion distance computation based on Tanimoto similarity. Initially, we compared the MoGA-T algorithm without acceptance probabilities with NSGA-II. The data in Table 2 and Table 3 show that the algorithm’s hypervolume and success rates are improved with the Tanimoto similarity-based congestion distance computation. Notably, an 11% increase in success rate was observed when optimizing the Osimertinib task, which may be attributed to the method’s ability to better highlight structural differences between molecules, proving its effectiveness. Subsequently, MoGA-T was compared with MoGA-TA. Table 2 and Table 3 show that there is a significant improvement in all optimization tasks after implementing the probabilistic acceptance strategy. This improvement is attributed to the fact that acceptance probabilities allow underperforming individuals to remain in the population, thus maintaining diversity, preventing premature convergence to local optima, and providing breeding opportunities for less adaptable individuals.

## 3. Materials and Methods

### 3.1. Related Work

Evolutionary algorithms play an important role in multi-objective drug molecule optimization, especially when dealing with multiple conflicting objectives and complex chemical spaces. In order to better introduce the method proposed in this paper, this section will first review the basic concepts and applications of drug molecule optimization and genetic algorithms. Through the discussion of existing methods, we will further elucidate the current status of practical applications of multi-objective optimization in drug molecule design, as well as the development trends and challenges in this field.

#### 3.1.1. Non-Dominated Sorting Genetic Algorithm II (NSGA-II)

The non-dominated sorting genetic algorithm II (NSGA-II) is an evolutionary algorithm for solving multi-objective optimization problems proposed by Deb et al. in 2002 [28]. The algorithm is able to efficiently solve optimization problems with multiple conflicting objectives through the mechanism of non-dominated sorting and congestion comparison. The core idea of NSGA-II is to simulate the process of natural selection through the operations of selection, crossover, and mutation, and thus to generate progressively more optimal solutions in the population. In the working principle of NSGA-II, the individuals in the population are firstly sorted into non-dominated orderings and categorized into multiple frontier tiers based on dominance relationships. Subsequently, the crowding distance is used to measure the distribution of individuals in each level to maintain the diversity of population distribution and avoid all solutions from clustering in a certain area. The pseudo-code is shown in Algorithm 1 [28,33]. In the field of drug molecule optimization, NSGA-II has been widely used in drug design, molecule optimization, and property prediction [38]. For example, methods such as graph-based elite patch illumination algorithm (GB-EPI) [35] and graph-based molecular Pareto optimization [33] have shown good performance in drug molecule optimization.

#### 3.1.2. Multi-Objective Molecular Optimization

The multi-objective molecular optimization is of great significance in the field of drug design and molecular engineering, involving the optimization of multiple, often conflicting, objectives, such as the biological activity, toxicity, solubility, stability, and pharmacokinetic properties of drug molecules [1,2,39]. Due to the intrinsic competition between these objectives, single-objective optimization methods make it difficult to take into account the needs of multiple objectives, and therefore, multi-objective optimization methods have become an inevitable choice [20,21]. The Pareto-based methodology is pivotal in multi-objective molecular optimization. It delineates the balance among objectives within the solution set through the “non-domination” principle. Utilizing Pareto optimal solution sets, it seeks to generate a collection of solutions that attain a relative equilibrium across objectives. These sets form Pareto frontiers, symbolizing optimal trade-offs between various objectives. The primary objective is to identify solution sets proximate to the Pareto frontier, leveraging optimization algorithms to achieve equilibrium among multiple objectives.
**Algorithm 1** The framework of the algorithm for NSGA-II.**Require:** 
*P* (initial population), *N* (number of generations)**Ensure:** 
$P_{final}$ (pareto front)1:fronts⇐frontssorting(P)2:crowding_distance⇐crowdingdistance(fronts)3:**for** i=0 to *N* **do**4:    $P_{i}^{\prime }⇐mutation\left(P_{i}\right)+crossover\left(P_{i}\right)$5:    $fronts⇐fronts\;sorting(P_{i}^{\prime }+P_{i})$6:    crowding_distance⇐crowdingdistance(fronts)7:    $P_{i+1}⇐Select\;satisfy\;conditions\left(\right)$8:**end for**

Currently, the commonly used evolutionary algorithms for multi-objective molecular optimization include the non-dominated sorting genetic algorithm II (NSGA-II), the conformational space annealing algorithm (MolFinder), etc. [23,28]. These algorithms ensure diversity and comprehensive exploration within the target space, thereby preventing the risk of converging to suboptimal solutions. This capability facilitates efficient navigation through chemical space. Given the escalating demands for precision and efficiency in drug design, enhancing the optimization strategy is crucial. By refining evolutionary algorithms and exploring superior methodologies, the efficacy and performance of multi-objective molecular optimization can be significantly improved. These advanced strategies enable more effective exploration of chemical space, effectively manage the trade-offs between various objectives, and augment both the diversity and quality of the solution set, ultimately aligning with the requirements of drug design for molecular generation and optimization [31].

In conclusion, multi-objective molecular optimization holds significant value in drug discovery and material design. As optimization algorithms advance and computational power improves, this method will increasingly influence molecular design and optimization, thereby accelerating the discovery of new drugs and enhancing the efficiency of molecular synthesis.

#### 3.1.3. Crossover and Mutation Operations

The genetic algorithm (GA) represents a traditional optimization technique grounded in principles of natural selection and genetic processes. In GA, crossover and mutation operations are pivotal for generating new individuals, preserving population diversity, and enhancing the quality of offspring. This study employed a decoupled crossover and mutation strategy to achieve more efficient molecular optimization. The crossover strategy significantly expands the exploration of the chemical space by decomposing the parent molecule into core structures and side-chain components, which are then randomly recombined. During the mutation process, different offspring molecules are obtained by adding, replacing, or deleting the side chains of the molecules [33]. The above crossover mutation strategy not only significantly improves the algorithm’s ability to explore the solution space but also enhances its adaptability and the quality of the generated molecules, thereby more effectively discovering high-quality solutions during the optimization process [19,32,37].

### 3.2. Method

This section introduces a multi-objective optimization approach utilizing Tanimoto crowding distance and acceptance probability (MoGA-TA) to generate and evaluate compounds meeting multi-objective optimization criteria. The framework integrates essential operations, including Tanimoto-based crowding degree, crossover, and mutation, to sustain population diversity and convergence while enhancing the success rate of molecular optimization in multi-objective tasks. Additionally, a dynamic acceptance probability mechanism, adjusted according to the number of generations, is implemented to maintain a balance between diversity and convergence throughout the evolutionary process. Subsequently, the multi-objective molecular optimization problem is outlined, followed by a detailed description of the methodological framework’s primary components and processes.

#### 3.2.1. Problem Definition

The optimization of lead compounds within the drug discovery and development process constitutes a multi-objective optimization challenge focused on enhancing various critical molecular properties. This process can be structured as an optimization task aimed at identifying the optimal molecular configuration, with each molecular characteristic serving as an objective for optimization. Starting from an initial lead compound, the objective is to navigate the chemical landscape to discover novel molecular structures that enhance multiple target properties. These properties may encompass elevated biological activity, improved selectivity, superior pharmacokinetic characteristics, and diminished toxicity. Mathematically, this multi-objective optimization problem can be defined as in Equation (2):

(2) $$max_{x\in \Omega }\left(f_{1}\left(x\right),f_{2}\left(x\right),\ldots ,f_{n}\left(x\right)\right)$$

where *x* denotes a molecule, Ω denotes the chemical space consisting of all molecules, and $f_{1}\left(x\right),f_{2}\left(x\right),\ldots ,f_{n}\left(x\right)$ denotes the *n* objectives to be optimized, each representing a particular molecular property. The solution to this problem is usually a collection of multiple molecular structures, known as a Pareto front. Each molecular structure provides a different trade-off between multiple objectives. The molecular properties to be considered for a typical situation include the following:

- Chemical rationality assessment: including synthetic accessibility (SA score), drug similarity (QED), LogP (lipid solubility), etc.
- Total polar surface area (TPSA): used to predict the ability of a molecule to cross a cell membrane [39].
- Molecular structure characteristics: including the number of aromatic rings (Number of Aromatic Rings), the number of rotatable bonds (Number of Rotatable Bonds), etc.
- Bioactivity: refers to the ability of a molecule to interact with a biological target (such as an enzyme, receptor, or ion channel).
- Similarity: The similarity score with the target molecule, usually calculated using Tanimoto similarity (based on different fingerprint types, such as FCFP4, ECFP6, etc.) [40]. Tanimoto similarity was calculated using Equation (3) below:

(3) $$Sim\left(x,x_{0}\right)=\frac{2\times |fp\left(x\right)\cap fp\left(x_{0}\right)|}{|fp\left(x\right)|+|fp\left(x_{0}\right)|\;-\;|fp\left(x\right)\cap fp\left(x_{0}\right)|}$$

where fp(x) and $fp(x_{0})$ represent the Morgan fingerprints of molecules *x* and $x_{0}$, respectively [41], and |·| denotes the size of the set, i.e., the number of elements in the set. This formula reflects the degree of overlap between the structural features of two molecules and is a value between 0 and 1. The closer the value is to 1, the higher the similarity. In this study, we used the RDKit toolkit to generate Morgan fingerprints and calculate Tanimoto similarity [35].

#### 3.2.2. Framework of MOGA-TA

Algorithm 2 delineates the primary framework of the proposed multi-objective optimization algorithm, MoGA-TA. The algorithm consists of the following steps: first, a population of size N is randomly selected from the specified dataset, and its fitness is calculated. Subsequently, offspring are generated through crossover and mutation operations, and their fitness is calculated. Next, the parent and child molecules are merged, Pareto sorting is used to obtain the Pareto front, and the crowding distance of the molecules in the Pareto front is calculated based on the Tanimoto distance. Finally, for frontiers requiring selection operations, they are sorted in descending order by crowding distances, and molecules entering the next generation are determined based on acceptance probability. This evolutionary process continues until the termination condition is met. The selection procedure integrates crowding distance sorting and acceptance probability to ensure the next generation of molecules is selected based on fitness and diversity metrics, thereby preserving population diversity. Consequently, the algorithm achieves a balance between exploration and exploitation, enhancing the likelihood of discovering a globally optimal solution. Evaluation metrics, including success rate, dominant hypervolume, geometric mean, and internal similarity, are employed to assess the overall optimization quality and population diversity. The fundamental process of the algorithm resembles that of the classical NSGA-II method, with enhancements in crowding distance calculations and dynamic acceptance probabilities based on Tanimoto similarity. Further details on other key components of MoGA-TA will be provided subsequently.
**Algorithm 2** The framework of the algorithm for MoGA-TA**Require:** 
*P* (initial population), *N* (number of generations)**Ensure:** 
Optimized molecules1:**for** i=0 to *N* **do**2:    $P_{i}^{\prime }⇐mutation\left(P_{i}\right)+crossover\left(P_{i}\right)$3:    $fronts⇐fronts\;sorting(P_{i}^{\prime }+P_{i})$4:    crowding_dis⇐crowding_distance(fronts)5:    accept_prob⇐acceptance_probability(i)6:    $P_{i+1}⇐\left[\right]$7:    **for** each front in fronts **do**8:        **if** satisfy_splitting_condition(front) **then**9:           $P_{i+1}⇐sort\_selection\left(front,accept\_prob\right)$10:        **else**11:           $P_{i+1}⇐P_{i+1}+front$12:        **end if**13:    **end for**14:**end for**

#### 3.2.3. Crowding Distance Based on Tanimoto Similarity

In NSGA-II (non-dominated sorting genetic algorithm II), the crowding distance is used to distinguish individuals within the Pareto front. In traditional NSGA-II, only the objective function value is usually considered when calculating the crowding distance. The solutions in the same non-dominated front are sorted in each objective dimension, and the normalized difference between adjacent individuals in this dimension is used to estimate the local density (sparseness), and finally, the difference in all dimensions is linearly accumulated as the crowding degree. However, this calculation method does not take into account the chemical structure information of the molecule and ignores the diversity of molecular structure. However, this computational method does not take into account the chemical structure information of the molecule, ignores the diversity of molecular structures, and focuses only on the objective function value. In drug design, the chemical structure of a molecule is pivotal, particularly when the objective function is indifferent to structural variations, potentially resulting in structural homogenization. The Tanimoto similarity-based crowding distance effectively reflects molecular structural similarity regardless of the objective function value. This method enhances the identification of structural differences among molecules, thereby fostering the generation of diverse molecular structures. In drug discovery, diverse molecular libraries significantly boost the likelihood of identifying effective drugs. In molecular optimization tasks, molecular structure (represented by SMILES) and chemical properties (such as similarity and functional group distribution) are paramount. Tanimoto similarity directly quantifies structural differences, aiding in the retention of structurally diverse molecules within the population. In particular, during the initial molecular screening phase, maintaining diversity in the molecular library increases the probability of discovering novel and effective compounds. During the optimization process, this approach is able to generate richer candidate molecules while optimizing by directly considering structural differences. In this case, the distance between pairs of molecules is calculated using the Tanimoto distance [33], which is defined as shown in Equation (4):

(4) $$d_{ij}=d\left(s_{i},s_{j}\right)=1-\frac{|s_{i}\cap s_{j}|}{|s_{i}\cup s_{j}|}$$

where $|s_{i}\cap s_{j}|$ denotes the number of common sites in the fingerprints of molecules $s_{i}$ and $s_{j}$, and $|s_{i}\cup s_{j}|$ denotes the number of all sites present in both molecular fingerprints (the fingerprint used in this paper is ECFP4). Based on the distances between pairs of molecules, we calculated the average distance between each molecule and all other molecules as a measure of crowding. By calculating the crowding distance in conjunction with Tanimoto similarity, the diversity and structural features of molecules can be evaluated more efficiently in multi-objective optimization.

#### 3.2.4. Population Update

To ensure the evolution of the population towards higher-quality candidate molecules, MOGA-TA employs a selection mechanism that integrates Tanimoto similarity-based crowding distances, Pareto ranking, and acceptance probabilities. After merging the parent and child generations, the merged molecules are first non-dominated sorted, and the crowding distance is calculated. Then, the contemporary acceptance probability is calculated according to Equation (5). Molecules are selected based on dominance rank and crowding, and included in the next iteration’s parent population using acceptance probability. During crossover and mutation, parent individuals are selected via random sampling based on fitness values. For mutation, individuals with higher fitness values have a higher likelihood of selection. For crossover, a comprehensive evaluation based on fitness values ensures that higher-fitness individuals are more frequently chosen. This selection process considers individual fitness while maintaining diversity through randomness, thereby mitigating the risk of premature convergence. The acceptance probability strategy effectively balances exploration and exploitation, preventing premature convergence to local optima by introducing randomness in the selection process. Early in the algorithm, a higher degree of exploration is permitted, allowing for diverse individuals. As evolution progresses, the focus shifts to selecting superior individuals, gradually converging towards the optimal solution. Each molecule has a probability of being selected for the next generation, though elimination is also possible. The acceptance probability formula is provided (5):

(5) $$p_{a}=e^{-(1/g)\beta }$$

where *g* denotes the number of current iteration generations, $p_{a}$ denotes the probability that the current molecule is accepted, and β controls the rate of decay, which is 0.45 in this paper. A smaller value of β implies a slower increase in the acceptance probability, while a larger value of β approaches 1 more quickly. Finally, this population renewal strategy maintains the diversity of the population during generation and selection while utilizing the good individuals that are already available. This balance helps to achieve better convergence and diversity.

## 4. Conclusions

This study transforms the optimization problem of lead drug molecules into a multi-objective optimization challenge. A novel optimization method, MoGA-TA, is introduced as a population updating strategy that utilizes crowding distance and dynamic acceptance probability adjustment based on Tanimoto similarity. The crowding calculation strategy in MoGA-TA helps capture the structural differences between molecules, thereby generating diverse molecular structures that are critical to improving the probability of drug discovery. The method improves selection accuracy by standardizing crowding treatment, thereby achieving fair comparison at different scales. In addition, robust management of invalid fingerprints and efficient computation on large datasets enhance the adaptability and computational efficiency of the algorithm. Together, these features promote a balance between exploration and exploitation, providing an effective strategy for solving complex molecular optimization problems. Dynamic acceptance probability adjustment in the population updating strategy ensures a balance between exploration and exploitation in the multi-objective optimization framework. By regulating the probability of individuals with poor fitness entering the next generation, the method helps maintain diversity while achieving convergence and effectively explores a wider solution space.

This study demonstrates the effectiveness of MoGA-TA in solving the multi-objective drug molecule optimization challenge. The method successfully explored a wider range of chemical fields, generated diverse molecules with different similarities, and optimized multiple objectives. Nevertheless, when faced with more optimization targets or more complex optimization problems, how to generate candidate molecules with better diversity and more ideal properties still needs further improvement. Therefore, there are still many research directions in the future, such as an in-depth study of the evolutionary process to generate more diverse, more widely distributed, and more ideal candidate molecules in the chemical space. In addition, improved cross-mutation technology is expected to bring more suitable drug molecule optimization strategies.

## Figures and Tables

**Table 1:** Multi-objective optimization benchmarks and corresponding scoring and modification functions.

Benchmark Name	Scoring Functions	Modifier
Fexofenadine	Tanimoto (AP)	Thresholded (0.8)
	TPSA	MaxGaussian (90, 10)
	logP	MinGaussian (4, 2)
Pioglitazone	Tanimoto(ECFP4)	Gaussian (0, 0.1)
	Molecular weight	Gaussian (356, 10)
	Number of rotatable bonds	Gaussian (2, 0.5)
Osimertinib	Tanimoto(FCFP4)	Thresholded (0.8)
	Tanimoto(ECFP6)	MinGaussian (0.85, 2)
	TPSA	MaxGaussian (95, 20)
	logP	MinGaussian (1, 2)
Ranolazine	Tanimoto (AP)	Thresholded (0.7)
	TPSA	MaxGaussian (95, 20)
	logP	MaxGaussian (7, 1)
	Number of fluorine count	Gaussian (1, 1)
Cobimetinib	Tanimoto(FCFP4)	Thresholded (0.7)
	Tanimoto(ECFP6)	MinGaussian (0.75, 0.1)
	Number of rotatable bonds	MinGaussian (3, 1)
	Number of aromatic rings	MaxGaussian (3, 1)
	CNS (0.5)	—
DAP kinases	DAPk1	Thresholded (0.8)
	DRP1	Thresholded (0.8)
	ZIPk	Thresholded (0.8)
	QED	Gaussian (0.8, 0.1)
	logP	MaxGaussian (3, 1)

**Table 2:** Supervolume, success rate, maximum geometric mean, and internal similarity results for methods NSGA-II, GB-EPI, and MOGA-TA in six multi-optimization tasks.

Algorithm	Task	HV	Success Rate	Geometric Mean	Internal Similarity
GB-EPI	Fexofenadine	0.67	0.33	0.87	0.50
	Pioglitazone	0.98	0.55	0.99	0.50
	Osimertinib	0.54	0.28	0.85	0.50
	Ranolazine	0.46	0.31	0.81	0.50
	Cobimetinib	0.77	0.60	0.93	0.50
	DAP kinases	0.04	0.17	0.50	0.51
NSGA-II	Fexofenadine	0.78	0.42	0.92	0.52
	Pioglitazone	1.00	0.64	1.00	0.51
	Osimertinib	0.66	0.33	0.89	0.52
	Ranolazine	0.68	0.36	0.87	0.51
	Cobimetinib	0.94	0.70	0.94	0.51
	DAP kinases	0.04	0.23	0.48	0.51
MoGA-TA	Fexofenadine	0.85	0.51	0.94	0.51
	Pioglitazone	1.00	0.73	1.00	0.50
	Osimertinib	0.70	0.52	0.90	0.52
	Ranolazine	0.75	0.42	0.89	0.51
	Cobimetinib	0.96	0.73	0.94	0.50
	DAP kinases	0.06	0.32	0.51	0.50

**Table 3:** Supervolume, success rate, maximum geometric mean, and internal similarity results for MoGA-TA without acceptance probability case.

Algorithm	Task	HV	Success Rate	Geometric Mean	Internal Similarity
MoGA-T	Fexofenadine	0.81	0.46	0.93	0.51
	Pioglitazone	1.00	0.67	1.00	0.50
	Osimertinib	0.68	0.44	0.89	0.51
	Ranolazine	0.71	0.35	0.88	0.51
	Cobimetinib	0.94	0.71	0.95	0.51
	DAP kinases	0.05	0.26	0.49	0.50

## Data Availability

Data is contained in the paper.
